# Circadian clocks in health and disease: Dissecting the roles of the biological pacemaker in cancer

**DOI:** 10.12688/f1000research.128716.2

**Published:** 2023-05-16

**Authors:** Bridget M. Fortin, Alisa L. Mahieu, Rachel C. Fellows, Nicholas R. Pannunzio, Selma Masri

**Affiliations:** 1 Department of Biological Chemistry, University of California, Irvine, Irvine, California, 92697, USA; 2 Department of Medicine, University of California, Irvine, Irvine, California, 92697, USA

**Keywords:** circadian clock, cancer, night shift work, early-onset cancer, colorectal cancer, Wnt signaling, chronotherapy, chronomedicine

## Abstract

In modern society, there is a growing population affected by circadian clock disruption through night shift work, artificial light-at-night exposure, and erratic eating patterns. Concurrently, the rate of cancer incidence in individuals under the age of 50 is increasing at an alarming rate, and though the precise risk factors remain undefined, the potential links between circadian clock deregulation and young-onset cancers is compelling. To explore the complex biological functions of the clock, this review will first provide a framework for the mammalian circadian clock in regulating critical cellular processes including cell cycle control, DNA damage response, DNA repair, and immunity under conditions of physiological homeostasis. Additionally, this review will deconvolute the role of the circadian clock in cancer, citing divergent evidence suggesting tissue-specific roles of the biological pacemaker in cancer types such as breast, lung, colorectal, and hepatocellular carcinoma. Recent evidence has emerged regarding the role of the clock in the intestinal epithelium, as well as new insights into how genetic and environmental disruption of the clock is linked with colorectal cancer, and the molecular underpinnings of these findings will be discussed. To place these findings within a context and framework that can be applied towards human health, a focus on how the circadian clock can be leveraged for cancer prevention and chronomedicine-based therapies will be outlined.

## Introduction

Biological rhythms regulate daily, seasonal, and long-term oscillations that are essential for life on Earth. The circadian clock is an evolutionarily conserved pacemaker found in prokaryotes and eukaryotes that governs homeostatic circuits that are fundamentally required for host fitness and survival. The mammalian clock is functionally conserved to regulate sleep/wake cycles ( Czeisler *et al*., 1980; Winfree, 1983), feeding/fasting rhythms ( Damiola *et al*., 2000; Hara *et al*., 2001; Inoue *et al*., 1977; Stokkan *et al*., 2001; Vollmers *et al*., 2009), and a host of endocrine, metabolic, and immune functions ( Green *et al*., 2008; Keller *et al*., 2009; Kitchen *et al*., 2020; Turek *et al*., 2005). The focus of this review is on mammalian clocks and their roles in health and disease, with a particular focus on clocks in healthy versus transformed cells. Recent evidence has cited multiple diverse and tissue-specific functions of the circadian clock in different cancer types such as lung, colorectal, hepatocellular, breast, and others ( Chun, Fortin, Fellows *et al*., 2022; Dong *et al*., 2019; Janich *et al*., 2011; Lee *et al*., 2010; Papagiannakopoulos *et al*., 2016; Puram *et al*., 2016; Stokes *et al*., 2021).

Notably, circadian regulation can occur not only at the level of the transcriptional and translational feedback loop (TTFL), but also through protein-based mechanisms ( Gotoh *et al.*, 2014; Huber *et al.*, 2016). This review will provide a comprehensive overview of the divergent functions of the clock in cell cycle control, maintenance of genome integrity, and immunity in healthy tissues, in an attempt to deconvolute the elaborate cellular networks that the biological pacemaker impinges on. Additionally, the reported role of the circadian clock in different cancer types will be reviewed in the context of clinical and epidemiology data, pre-clinical *in vivo* mouse models, as well as mechanistic data from cell line-based studies. By considering the tissue- and cell-specific roles of the circadian clock as transcriptional regulators as well as at the protein level, this review will provide a comprehensive and updated understanding of the intriguing connections between the circadian clock and cancer biology.

## Clocks in healthy tissues

**Figure 1.**
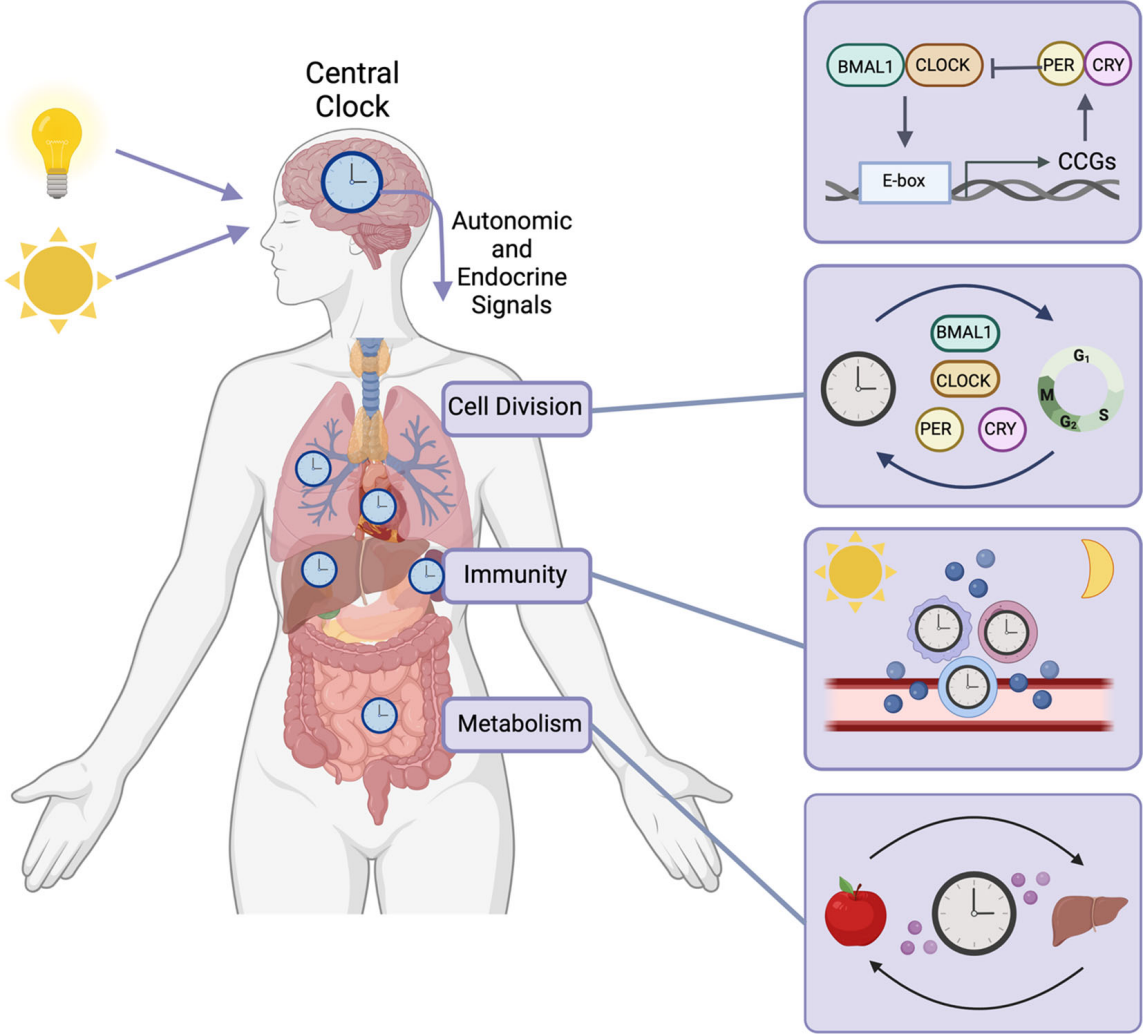
Circadian clock function in normal tissues. In mammals, circadian rhythms are coordinated by the central circadian clock, located in the suprachiasmatic nucleus (SCN) of the hypothalamus ( Inouye & Kawamura, 1979; Stephan & Zucker, 1972). The central clock receives photic cues and transmits endocrine and autonomic signals to synchronize tissue-specific peripheral clocks to the time of day ( Pando *et al*., 2002; Welsh *et al*., 2004, 2010; Whitmore *et al*., 2000; Yamazaki *et al*., 2000). The circadian clock is regulated by a TTFL where CLOCK and BMAL1 drive transcriptional activation and PERIOD (PER) and CYPTOCHROME (CRY) feedback to repress this transcriptional activity. This TTFL regulates gene expression programs that modulate critical cellular processes needed to maintain homeostasis including cell division, maintenance of genome integrity, immunity, endocrine and metabolic functions. The circadian clock is implicated in regulating the growth and division of cells as the expression of cyclins is rhythmic ( Graña & Reddy, 1995; Vermeulen *et al*., 2003). Circadian proteins also mediate the DNA damage response ( Gery *et al*., 2006; Kang & Leem, 2014) and DNA repair including nucleotide excision repair ( Gaddameedhi *et al*., 2011; Marteijn *et al*., 2014), base excision repair ( Kozmin *et al*., 2005; Krokan & Bjørås, 2013), homologous recombination and non-homologous end-joining ( Cotta-Ramusino *et al*., 2011; Shafi *et al*., 2021). Importantly, in addition to its transcriptional regulation, the circadian clock also exerts its function at the protein-level, with PER2 directly binding to inhibit p53 degradation ( Gotoh *et al.*, 2014) and CRY2 promoting MYC degradation ( Huber *et al.*, 2016). In addition to regulation of cell division and DNA damage, the immune system is also tightly regulated by the circadian clock to promote efficient immunologic response to infection. Immune cells have functional circadian clocks ( Keller *et al*., 2009; Silver *et al*., 2012) and the release of cytokines and chemokines is rhythmic ( Gibbs *et al*., 2014; Pariollaud *et al*., 2018), as well as the release of immune cells into the bloodstream ( Dimitrov *et al*., 2007; Méndez-Ferrer *et al*., 2008). This rhythmic secretion of chemokines facilitates time of day trafficking of immune cells into tissues ( Gibbs *et al*., 2014; Méndez-Ferrer *et al*., 2008) which has been demonstrated to mediate the host response to infection ( Kiessling *et al*., 2017) and disease ( Gibbs *et al*., 2014; Kitchen *et al*., 2020). Lastly, metabolic processes, including glucose and lipid metabolism, cardiovascular health and endocrine hormone secretion are regulated by the circadian clock ( Green *et al*., 2008; Verlande & Masri, 2019). Figure created using BioRender.

The circadian clock is the internal biological pacemaker that controls cell autonomous 24-hour oscillations in gene expression programs that regulate organismal physiology ( Figure 1). The central clock, which resides in the suprachiasmatic nucleus (SCN) of the hypothalamus, is responsive to photic cues and transmits autonomic and endocrine signals to synchronize tissue-specific peripheral clocks to the environmental light-dark cycle ( Pando *et al*., 2002; Welsh *et al*., 2004, 2010; Whitmore *et al*., 2000; Yamazaki *et al*., 2000). Peripheral clocks are also entrained by external cues including temperature ( Barrett & Takahashi, 1995; Brown *et al*., 2002; Gould *et al*., 2006; Huang *et al*., 1995; Ruoff *et al*., 2005) and food supply that serve to further fine-tune biological timekeeping ( Damiola *et al*., 2000; Hara *et al*., 2001; Inoue *et al*., 1977; Stokkan *et al*., 2001; Vollmers *et al*., 2009). The circadian system is regulated by a tightly controlled TTFL that encompasses a 24-hour day. The positive transcriptional activators of the circadian machinery, CLOCK and BMAL1, heterodimerize and bind to consensus E-box motifs located within promoters of core clock and clock-controlled genes ( Ripperger & Schibler, 2006; Ueda *et al*., 2005). The core clock regulators of the negative arm of this TTFL, PERIOD (PER) and CRYPTOCHROME (CRY), are translated to repress the transcriptional activity of the CLOCK-BMAL1 complex ( Duong *et al*., 2011; Michael *et al*., 2017; Nangle *et al*., 2014; Narasimamurthy *et al*., 2018). This entire transcriptional/translational feedback circuit drives the rhythmic periodicity of gene expression networks that govern endocrine function, metabolism, immune response, cell cycle control, and genome stability, many of which will be discussed below. Given the complex role of the clock in cellular metabolic control and nutritional challenge, and the host of recent publications covering this topic ( Guan & Lazar, 2021; Reinke & Asher, 2019; Rijo-Ferreira & Takahashi, 2019; Verlande & Masri, 2019), this review will only cover clock-controlled metabolic alterations in the context of cancer.

### Linking cell cycle checkpoints with the clock

Cells regulate their growth and division using cell cycle checkpoints, which ensure timely progression of the cell cycle under normal physiological conditions, and also halt cell cycle progression in instances of DNA damage, erroneous mitosis, or environmental stressors ( Collins *et al*., 1997; Kastan & Bartek, 2004). The duration and transition of each cell cycle phase is orchestrated by the activation of specific cyclin-dependent kinases (CDKs) by their respective cyclins ( Graña & Reddy, 1995; Vermeulen *et al*., 2003). Whereas expression of CDKs remains relatively constant throughout the cell cycle, each cyclin peaks in expression in a staggered and coordinated manner to drive the cell cycle with appropriate timing ( Graña & Reddy, 1995; Vermeulen *et al*., 2003). Given the rhythmic nature of both the cell cycle and the circadian clock, several studies have worked to investigate and define the connections between these two systems. It has been suggested that circadian rhythms and the cell cycle are tightly phase-coupled and oscillate with a 1:1 frequency in mouse fibroblasts ( Feillet *et al*., 2014), and this synchronization has even been shown at the single cell level in mammalian NIH3T3 fibroblasts ( Bieler *et al*., 2014). More recently, data suggests that the cell cycle and the circadian clock can synchronize each other bidirectionally in mammalian systems ( Yan & Goldbeter, 2019). However, the extent of this coupling is still not well understood, as some studies report additional findings that the cell cycle and circadian clock can in fact operate independently, as demonstrated in Rat-1 fibroblasts as well as Lewis lung carcinoma cell lines ( Yeom *et al*., 2010; Pendergast *et al*., 2010).

Given the intimate links between the circadian clock and the cell cycle, what are the molecular connections driving this interplay? Importantly, several studies have investigated connections between the circadian clock and the proto-oncogene MYC, a master regulator of cell cycle control that promotes cellular growth by driving cyclin expression and repressing CDK inhibitor activity ( Burchett *et al*., 2021). In regard to transcriptional regulation of *c-Myc *, it has been reported that *Bmal1 ^-/-^* mice exhibited increased expression of *c-Myc *, whereas *c-Myc* expression was decreased in *Cry1 ^-/-^;Cry2 ^-/-^* mice ( Liu *et al*., 2020), demonstrating a strong correlation between these networks. Additionally, MYC and NMYC can impinge on circadian rhythms in U2OS and SHEP cells, respectively, further highlighting the molecular crosstalk between the clock and the cell cycle that also integrates with cellular metabolic state ( Altman *et al*., 2015; Shostak *et al*., 2016; Moreno-Smith *et al.*, 2021). In regard to regulation of MYC protein levels, it was demonstrated that CRY2 mediates MYC degradation and that *Cry2 ^-/-^* knockout induced increased proliferation and transformation rates ( Huber *et al*., 2016).

In addition to circadian links to MYC, clock-dependent transcription has been observed for many other cell cycle modulators, including cyclin D, cyclin E, cyclin A, and cyclin B in human epithelium ( Bjarnason *et al*., 1999; Fu *et al*., 2002). Likewise, G2/M regulator WEE1, which inhibits Cyclin B1-CDK1 activity, has oscillating protein expression and kinase activity and this oscillation was dampened in *Cry1 ^-/-^;Cry2 ^-/-^* mice ( Gréchez-Cassiau *et al*., 2008; Matsuo *et al*., 2003). Another study found that *CLOCK* and *BMAL1* knockdown leads to the suppression of *WEE1* and thus an increased activation of apoptosis in human hepatocellular carcinoma cell lines, again confirming circadian influence on WEE1 regulation ( Qu *et al.*, 2023). Additional CDK inhibitors such as p21 ^cip1/waf1^ and p16-Ink4A also oscillate under circadian control and this rhythmicity was lost in *Bmal1 ^-/-^* mice or *Per1 ^Brdm1/Brdm1^;Per2 ^Brdm1/Brdm1^* mutant mice, respectively ( Gréchez-Cassiau *et al.*, 2008; Kowalska *et al.*, 2013). Taken together, these data highlight the important molecular links between the circadian clock and cell cycle control mechanisms.

Due to a multitude of interactions between circadian proteins and cell cycle checkpoints and drivers, it is not surprising that circadian rhythm disruption can modify rates of cellular proliferation. For example, genetic clock disruption in mouse osteoblasts via *Per1 ^-/-^;Per2 ^m/m^* knockout or *Cry1 ^-/-^;Cry2 ^-/-^* knockout resulted in increased proliferation ( Fu *et al*., 2005). Circadian clock disruption can potentially interfere with normal rates of cellular growth, introducing susceptibility to disease and cancer. Altogether, this data suggests that oscillations of clock proteins contribute to the proper expression of important cell cycle regulators that impact cellular proliferation.

### Interplay between the clock and the DNA damage response

Another important aspect of cell cycle regulation features the DNA damage response (DDR). Individual cells can receive tens of thousands of DNA lesions per day, which leads to replication errors, transcription blockage, and even permanent mutations if left unrepaired ( Jackson & Bartek, 2009). Thus, the DDR has evolved to preserve genome integrity by recognizing various forms of DNA damage, stimulating DNA repair, inhibiting cell cycle progression until the repair is complete, and inducing apoptosis if the damage is irreparable ( Giglia-Mari *et al*., 2011; Jackson & Bartek, 2009; Roos & Kaina, 2013).

In mammals, central DDR proteins ATR and ATM instigate the DDR by phosphorylating CHK1 and CHK2, respectively ( Jackson & Bartek, 2009). Consequently, phosphorylated CHK1 and CHK2 activate transcription factor p53 to halt cell cycle progression ( Ronco *et al*., 2017). It was reported that circadian proteins CRY1 and TIM modulate ATR and CHK1 activity in a time-of day dependent fashion, although it was found that *Cry1 ^-/-^;Cry2 ^-/-^* mouse embryonic fibroblasts (MEFs) still retained appropriate levels of checkpoint activity ( Kang & Leem, 2014). Also, ATM and CHK2 form a complex with circadian protein PER1, and it was further demonstrated that PER1 knockdown reduced ATM-mediated CHK2 phosphorylation and dampened apoptotic response to DNA damage in human colon cancer cell line HCT116 ( Gery *et al*., 2006).

Furthermore, a bi-directional crosstalk between the circadian clock and p53 has been reported. The p53 tumor suppressor plays a key role in stimulating DNA damage checkpoints and a circadian oscillation of p53 transcription has been reported in human oral epithelium ( Bjarnason *et al*., 1999). Concurrently, it has been demonstrated that p53 modulates circadian activity in mice by directly binding to the promoter of *Per2* and repressing *Per2* expression ( Miki *et al*., 2013). Moreover, PER2 directly binds to the C-terminal end of human p53 and slows MDM2-mediated degradation of p53 ( Gotoh *et al*., 2014), indicating multiple points of interplay between these two mechanisms. The consequences of clock regulation on p53 were evident in *Per2 ^m/m^* mutant mice that exhibited lower levels of p53, increased resistance to p53-mediated apoptosis, and higher sensitivity to γ radiation ( Fu *et al*., 2002). p53 activates the CDK inhibitor p21 ^cip1/waf1^ to induce cell cycle arrest ( Al Bitar & Gali-Muhtasib, 2019). As stated in the previous section, p21 ^cip1/waf1^ expression oscillates under circadian control, further demonstrating clock regulation of the DDR. Interestingly, it was reported that either *Cry1 ^-/-^* or *Cry2 ^-/-^* MEFs exhibit altered expression patterns of the p21 ^cip1/waf1^ transcript, *Cdkn1a*, in response to genotoxic stress via doxorubicin ( Papp *et al*., 2015). This study further demonstrated that genotoxic stress can shift the CRY1/CRY2 ratio and consequently change circadian period length ( Papp *et al*., 2015), again demonstrating the crosstalk between the DDR and the molecular machinery of the circadian clock.

In summary, these studies suggest that circadian proteins exert a wide influence on proper cellular response to the daily insults of DNA damage. Impaired DDR via clock disruption increases the likelihood of cell proliferation despite unresolved mutations, which is a major contributor to cancer progression. Furthermore, since DDR proteins are commonly targeted during chemotherapy to inhibit rapidly dividing cells, understanding the effects of oscillating circadian proteins and their impact on the DDR may result in enhanced efficacy of cancer therapeutics.

### Clock regulation of DNA damage repair

The circadian clock not only plays a role in regulating DNA damage checkpoints, but also affects the ability of cells to perform DNA repair. Cells are equipped with multiple repair pathways that act to maintain DNA sequence fidelity following damage from endogenous and exogenous sources. Interestingly, while each DNA repair pathway has several components, the activities of certain key components exhibit striking transcriptional regulation through the circadian clock ( Sancar *et al*., 2010).

Nucleotide excision repair (NER) removes bulky chemical adducts that distort the DNA helix, most importantly UV-induced intrastrand crosslinks ( Marteijn *et al*., 2014). NER relies on the xeroderma pigmentosum group A (XPA) protein to recognize the lesion and coordinate incision and removal by the XPF-ERCC1 endonuclease complex ( Marteijn *et al*., 2014). It was recently described that XPA oscillates in a circadian fashion, thus repair activity after UV irradiation also follows circadian rhythmicity. However, this rhythmicity was lost in *Cry1 ^-/-^;Cry2 ^-/-^* mice, clearly demonstrating that NER activity is regulated by the clock ( Gaddameedhi *et al*., 2011). As sunlight is the major source of UV radiation, this connection between DNA repair of UV-induced damage and the circadian clock is intriguing.

Base excision repair (BER) uses a variety of different DNA glycosylases to remove damaged bases, leaving an abasic site that is subsequently processed by enzymes that carry out cleavage, gap-filling and ligation to restore DNA integrity ( Krokan & Bjørås, 2013). The expression and activity of one such glycosylase, 8-Oxoguanine DNA glycosylase (OGG1), oscillates under clock control and OGG1 levels were disrupted in a human cohort performing shift work ( Manzella *et al*., 2015). Importantly, this study was performed using lymphocytes collected from human blood whereas another study found that OGG1 does not oscillate in human keratinocytes ( Hettwer *et al.*, 2020). This suggests a potential cell-type specific role of OGG1 circadian regulation. As OGG1 recognizes a specific type of oxidative damage (i.e., 7,8-dihydro-8-oxoguanine opposite cytosine or thymine), it remains to be seen why this enzyme has evolved to be clock-controlled. One interesting link is that OGG1 has been shown to be required to prevent mutations induced by UVA ( Kozmin *et al*., 2005), suggesting an additional role of the clock in repairing DNA damage following sunlight exposure.

DNA double strand breaks (DSBs) represent one of the most serious threats to genome integrity and multiple repair pathways have evolved for their repair, including homologous recombination (HR) and non-homologous end joining (NHEJ) ( Chang *et al*., 2017; Sterrenberg *et al*., 2022). Due to the number of environmental and cellular sources of DSBs, understanding the role of the circadian clock in their repair is critical. Using HEK293T cells, CLOCK binding was found at several enhancer or transcriptional regulatory sites controlling DNA damage related genes including *CDKN1A* encoding p21, which mediates cell cycle arrest, as well as *BRCA1* and *RAD50*, which play important roles in DSB repair ( Alhopuro *et al.*, 2010). Furthermore, CLOCK knockdown in human U2OS osteosarcoma cells resulted in abnormal cell cycle checkpoint response following irradiation and increased sensitivity to mitomycin C, indicative of a CLOCK-dependent response to repair DSBs ( Cotta-Ramusino *et al*., 2011). CLOCK was also found to localize to laser-induced DSBs in U2OS cells, suggesting a potential direct role in the cellular signaling machinery required for DSB repair ( Cotta-Ramusino *et al.*, 2011). In addition to CLOCK, CRY1 is another circadian protein linked to DSB repair efficiency. *CRY1* knockdown showed delayed DSB resolution in C4-2 and 22Rv1 cell cultures, and conversely, DSB resolution is enhanced upon treatment with the CRY1 stabilizer KL001 ( Shafi *et al*., 2021). Transcriptomic analysis suggested that CRY1 regulated the expression of several major HR genes (including *RAD51*, *BRCA1*, and *BRCA2*) and other genes involved in NER, BER, mismatch repair (MMR), and NHEJ ( Shafi *et al*., 2021). This study was specifically carried out in human prostate cancer cell lines as well as tissues from prostate cancer patients, and further studies are needed to define the role of CRY1 in regulation of DNA repair genes in other tissues, particularly those that are not hormone responsive.

Overall, these studies show that the effect of the circadian clock on DNA repair is widespread across multiple repair pathways. Although the mechanistic links continue to be investigated, current data suggests that robust circadian rhythms contribute to optimal genome integrity. Further understanding of how the circadian clock potentiates faithful DNA repair through multiple pathways is paramount for developing strategies to both prevent cancer, and to establish better and less toxic treatments for patients undergoing chemo- and radiation- therapy that acts through damaging DNA.

### The circadian clock regulates the immune response

The goal of the immune system is to be primed to respond to insult through a complex network of different organs, proteins and pathways. It may be advantageous for immune parameters to cycle with activity of an organism, potentially allowing for the host to respond more efficiently to infection. In support of this, the circadian clock has been shown to regulate key parameters of immunity including cytokine release, immune cell number and trafficking, as well as the inflammatory response.

Immune cells, including splenic macrophages, dendritic cells (DCs), and B cells have been found to have cell-autonomous circadian clocks which directly control cellular immune function and timing ( Keller *et al*., 2009; Silver *et al*., 2012). Cytokines and chemokines are small proteins that regulate the growth, activity and trafficking of immune cells and proper regulation of these proteins is essential for host immune defense. Importantly, the circadian clock has been linked to the production and release of cytokines and chemokines. Upon bacterial endotoxin stimulation, the secretion of TNFα and IL-6 by isolated *ex vivo* spleen derived macrophages was found to oscillate in a time-of-day dependent manner, including 8% of the macrophage transcriptome ( Keller *et al*., 2009). Additional studies demonstrated an important role for *Bmal1* in regulating cytokine response. Temporal gating of endotoxin-induced cytokine response in mice, a crucial feature of innate immunity, is dependent on the circadian clock as rhythmic gating of endotoxin response is lost in *Bmal1* ^-/-^ macrophages ( Gibbs *et al*., 2012). This was found to be due to the suppression of *Nr1d1*, hereafter referred to as *Rev-Erbα* ( Gibbs *et al*., 2012). An additional role of *Bmal1* in regulating the immune response was identified with the genetic ablation of *Bmal1* in bronchiolar cells that disrupted the rhythmic expression of the *CXCL5* chemokine ( Gibbs *et al*., 2014). These data suggest that the rhythmic release of cytokines is directly regulated by the circadian clock.

In addition to the rhythmic secretion of cytokines by immune cells, the circadian clock controls immune cell number and infiltration. For example, the number of hematopoietic stem cells (HSCs) and mature immune cells released from the bone marrow into the blood peaks at the beginning of the rest phase in mice ( Méndez-Ferrer *et al*., 2008). In addition to the release of immune cells into the bloodstream, the circadian clock also modulates immune cell trafficking into tissues as evidenced by the rhythmic expression of CXCL5 and CXCL12 that regulate the trafficking and infiltration of neutrophils and HSCs, respectively ( Gibbs *et al*., 2014; Méndez-Ferrer *et al*., 2008). Human studies provide additional evidence of circadian regulation of immune cell trafficking. Immune cells present in the blood of individuals on a normal sleep-wake cycle were compared to those on 24 hours of wakefulness ( Dimitrov *et al*., 2007). It was found that the number of DCs and T cells in the blood is highly rhythmic and that sleep induced the expression of IL-12 which increased the number of monocytic DCs in the blood ( Dimitrov *et al*., 2007). A more recent study found that individuals with blunted rest-activity rhythms exhibited increased inflammatory markers and elevated circulating white blood cells and neutrophils ( Xu *et al*., 2022). These studies demonstrate a clock-controlled immune response through regulation of immune cell release into the bloodstream and trafficking into tissues.

Clock control of the immune system is critical for proper response to infection ( Kiessling *et al*., 2017) and disease ( Gibbs *et al*., 2014; Kitchen *et al*., 2020), and even vaccination ( Cervantes-Silva *et al.*, 2022). In support of this, mice infected with *Salmonella enterica* in the early rest period exhibited a high pathogen load and a stronger proinflammatory response ( Bellet *et al*., 2013) and the magnitude of *Leishmania* parasitic infection in mice varied over 24 hours ( Kiessling *et al*., 2017). These differences in infection and inflammation may be due to the time-dependent release of cytokines and immune cells. Indeed, the circadian expression of chemo attractants and the rhythmic infiltration of neutrophils and macrophages was lost in clock deficient macrophages ( Kiessling *et al*., 2017; Sato *et al*., 2014). Additionally, pulmonary inflammation was found to be regulated by the rhythmic expression of the chemokine CXCL5 leading to time-of-day dependent neutrophil recruitment to the lung ( Gibbs *et al*., 2014). *Bmal1* ^-/-^ bronchiolar cells lack this rhythmic CXCL5 expression leading to exaggerated inflammatory response and an impaired host response to *Streptococcus pneumoniae* infection ( Gibbs *et al*., 2014). *Bmal1* deletion suppresses *Rev-erbα* expression, and it was found that *Rev-erbα ^-/-^* mice exhibit an exaggerated neutrophilic inflammatory response ( Pariollaud *et al*., 2018). Furthermore, myeloid specific deletion of *Bmal1* disrupts the diurnal trafficking of Ly6Hi inflammatory monocytes and promotes inflammation by inducing expression of monocyte attracting chemokines ( Nguyen *et al*., 2013).

Overall, these studies establish the circadian clock as a critical regulator of the immune response through the release of cytokines and the trafficking of immune cells. This leads to a time-of-day dependent proinflammatory response to challenge such as bacterial or pathogenic infection. Moreover, disruption of the circadian clock has the potential to alter the daily rhythm of the immune system and lead to various types of diseases, including cancer.

## Connecting the dots: The circadian clock and cancer

In the previous section, we described how the circadian clock regulates critical cellular processes including cell cycle control, the DNA damage response and repair, as well as immunity. These processes are included as ‘Hallmarks of Cancer’ that are dysregulated during transformation ( Hanahan, 2022; Hanahan & Weinberg, 2000, 2011), which suggests that the circadian clock may be involved in tumorigenesis. In support of this, we describe how the circadian clock is associated with cancer by looking at epidemiological data, early-onset cancers, and the tissue-specific and cell-type dependent function of the clock in various model systems.

### Epidemiological data: Night shift work and cancer risk

About one quarter of the US population participates in night shift work ( Alterman *et al*., 2013; Drake & Wright, 2017), which causes significant misalignment between the endogenous circadian clock and the sleep-wake cycle ( James *et al*., 2017). Night shift work has been implicated as a risk factor for cancer and a systematic review of night shift work and cancer was recently reported ( IARC, 2020). Several studies were identified that aimed to assess a correlation between night shift work and cancer risk, and these reports are summarized in Table 1. The most extensively studied association was between night shift work and breast cancer, and the majority of these reports found that night shift work increased the risk of developing breast cancer ( Cordina-Duverger *et al*., 2018; Hansen & Lassen, 2012; Jones *et al*., 2019; Schernhammer *et al*., 2006). Several studies also noted an increased risk with duration of exposure and cumulative exposure to night shift work ( Cordina-Duverger *et al*., 2018; Davis *et al*., 2001; Hansen & Lassen, 2012; Hansen & Stevens, 2012; Lie *et al*., 2011). In addition to breast cancer, shift work was also found to increase the risk of developing prostate, colon and rectum, lung, stomach, ovarian and pancreatic cancer. Although numerous studies cite a positive correlation between night shift work and cancer incidence, other studies report no effect ( Jones *et al*., 2019; Koppes *et al*., 2014; Li *et al*., 2015; O’Leary *et al*., 2006; Pronk *et al*., 2010; Travis *et al*., 2016; Vistisen *et al*., 2017). There are multiple explanations for the contradictory data, including the lack of a standardized definition for night shift work, self-reporting collection process, and adjustment for confounding factors such as socioeconomic status and lifestyle. These limitations should be addressed and larger, more comprehensive studies are needed with multiple cancer types to define the epidemiological link between the circadian clock and cancer risk.

**Table 1.** Night shift work and the risk of cancer.

Reference	Cancer type	Night shift work increases risk of cancer (yes/no)
( Tynes *et al*., 1996)	Breast	Yes, women over 50
( Lie *et al*., 2006)	Breast	Yes, with 30+ years
( Pronk *et al*., 2010)	Breast	No
( Lie *et al*., 2011)	Breast	Yes, with 5+ years, risk increased with duration of exposure and cumulative exposure
( Hansen & Stevens, 2012)	Breast	Yes, risk increased with duration of exposure and cumulative exposure
( Hansen & Lassen, 2012)	Breast	Yes, risk increased with duration of exposure and cumulative exposure
( Knutsson *et al*., 2013)	Breast	Yes
( Koppes *et al*., 2014)	Breast	No
( Li *et al*., 2015)	Breast	No
( Åkerstedt *et al*., 2015)	Breast	Yes, with 20+ years
( Travis *et al*., 2016)	Breast	No
( Wegrzyn *et al*., 2017)	Breast	Yes, with over 20+ years
( Vistisen *et al*., 2017)	Breast	No
( Jones *et al*., 2019)	Breast	No
( Schernhammer *et al*., 2001)	Breast	Yes
( Schernhammer *et al*., 2006)	Breast	Yes
( Cordina-Duverger *et al*., 2018)	Breast	Yes, risk increased with duration of exposure and cumulative exposure
( Pesch *et al*., 2010)	Breast	Yes
( Rabstein *et al*., 2013)	Breast	Yes
( Fritschi *et al*., 2013)	Breast	Yes
( Grundy *et al*., 2013)	Breast	Yes
( Menegaux *et al*., 2012)	Breast	Yes
( Cordina-Duverger *et al*., 2016)	Breast	Yes
( Papantoniou *et al*., 2016)	Breast	Yes
( Davis *et al*., 2001)	Breast	Yes, risk increased with duration of exposure and cumulative exposure
( O’Leary *et al*., 2006)	Breast	No
( Wang *et al*., 2015)	Breast	Yes
( Yang *et al*., 2019)	Breast	Yes
( Barul *et al*., 2019)	Prostate	Yes
( Wendeu-Foyet *et al*., 2018)	Prostate	Yes
( Papantoniou *et al*., 2017)	Colorectal	Yes
( Schernhammer *et al*., 2003)	Colorectal	Yes
( Gu *et al*., 2015)	Lung	Yes
( Schernhammer *et al*., 2013)	Lung	Yes
( Gyarmati *et al*., 2016)	Stomach	Yes
( Carter *et al*., 2014)	Ovarian	Yes
( Parent *et al*., 2012)	Pancreatic	Yes

### Early onset cancers and the circadian clock

The previous section highlighted the potential increase in cancer incidence in populations that participate in night shift work, which is known to disrupt circadian rhythms. However, there is mounting concern for circadian disruption in the general population as the access to technological devices continues to increase. Gradisar *et al*. demonstrated that nine out of 10 individuals surveyed use a technological device in the hour before bed, with the use increasing in individuals under 30 years of age ( Gradisar *et al*., 2013). Among the Japanese population, young adults between the ages of 15 to 20, were exposed to the highest intensity of artificial light-at-night ( Chen *et al*., 2022). The exposure to dim light at night through the use of devices has been shown to disrupt circadian rhythmicity by suppressing melatonin and impairing sleep quality ( Lee & Kim, 2019). This suggests that younger individuals may be exposed to more environmental factors that disrupt the circadian clock than older populations. Strikingly, the average annual increase in the incidence of all cancers in young adults aged 15 to 39 years old has continued to increase since 1975 ( Miller *et al*., 2020). A review of 98 articles published between 1995–2020 found that the incidence of colorectal, breast, kidney, pancreas, and uterine cancer is increasing in younger age groups ( di Martino *et al*., 2022). In addition to the increasing incidence, studies have also suggested that the underlying biology of cancer in young adults differs from the same cancer in children or older individuals ( Tricoli *et al*., 2016, 2018). Altogether, this introduces the idea that environmental circadian clock disruption in younger populations may contribute to the increasing incidence of early-onset cancers, though further studies are needed to confirm this experimentally.

It is worth noting that the increasing trends of early-onset cancers are strongest for colorectal cancer (CRC). Between 1975 and 2010, there has been a steady decline in CRC incidence rates in adults over the age of 50. However, in patients aged 20 to 34, the incidence of CRC has continued to rise ( Bailey *et al*., 2015). If this trend continues, it is expected that by 2030, the incidence of colon and rectal cancer in individuals aged 20 to 34 will increase by 90% and 124.2%, respectively ( Bailey *et al*., 2015). It was also identified that the increasing incidence of CRC in younger populations was greatest among Hispanics and African Americans, suggesting an alarming cancer health disparity ( Augustus & Ellis, 2018; Muller *et al*., 2021; Singh *et al*., 2014).

Importantly, the intestine may be particularly sensitive to circadian disruption due to several reasons. The intestine is a highly regenerative organ, with complete cell renewal occurring every few days ( Van Der Flier & Clevers, 2009). This constant renewal process is tightly regulated by the circadian clock, and disruption of circadian rhythms can affect the timing and coordination of this turnover process ( Codoñer-Franch & Gombert, 2018; Stokes *et al.*, 2017; Yoshida *et al.*, 2015). The gut microbiota, which play a crucial role in digestion and immune function, also exhibit circadian rhythmicity and can be negatively impacted by circadian disruption ( Heddes *et al.*, 2022; Leone *et al.*, 2015; Liang *et al.*, 2015; Thaiss *et al.*, 2016). Moreover, food intake is a powerful environmental cue synchronizing the circadian clock in peripheral tissues ( Zarrinpar *et al.*, 2014). Feeding mice only during the light phase, when mice are inactive, causes a phase shift in peripheral clocks of the liver, kidney, heart and pancreas ( Damiola *et al*., 2000; Stokkan *et al*., 2001), demonstrating that the timing of food intake can disrupt the circadian clock. Nutritional challenge has also been shown to dynamically impact the circadian clock, including high fat diet and time-restricted eating ( Acosta-Rodríguez *et al*., 2022; Chaix *et al*., 2019; Eckel-Mahan *et al*., 2013; Hatori *et al*., 2012). These studies establish the importance of coordinated cell renewal, gut microbiota, diet and timing of food intake in maintaining robust circadian rhythms in the intestine. It will be important to study the impact of alterations in these environmental and behavioral factors on the alarming increase in CRC rates in younger populations, as well as other cancer types.

### Circadian clock function in various tissues and model systems

**Figure 2.**
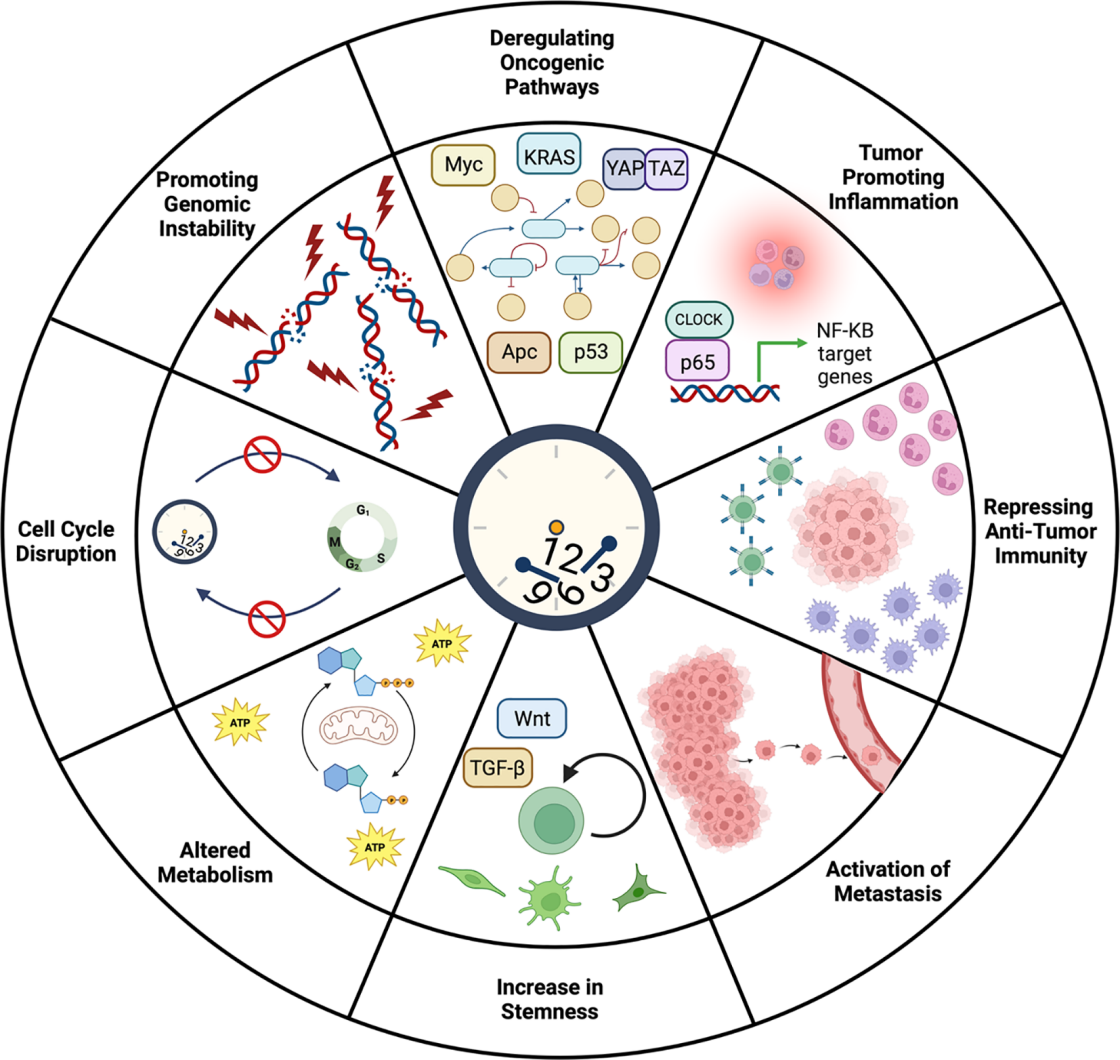
Potential roles of circadian clock disruption during tumor initiation and progression. In normal tissue, the circadian clock maintains homeostasis through diverse functions including control of the cell cycle, genome integrity, immunity, and metabolism. Given the numerous roles of the circadian clock in maintaining physiology, it is not surprising that the clock has been implicated in cancer initiation and progression. Indeed, a large body of evidence has linked the circadian clock to processes that become dysregulated during tumorigenesis including the cell cycle, proliferation, genome stability, stemness, metastasis, inflammation, immunity, and oncogenic signaling pathways. Analyzing over 32 different cancer types, it was found that clock genes are associated with activation or inhibition of oncogenic signaling pathways including phosphatidylinositol 3-kinase (PI3K)/AKT and RAS/mitogen-activated protein kinase (MAPK) signaling pathways ( Ye *et al*., 2018). Knockout of *Bmal1* was shown to accelerate *Apc* LOH in a mouse model of CRC suggesting that the clock may be involved in maintaining genome integrity ( Chun, Fortin, Fellows *et al*., 2022). With regards to the role of the clock in the cell cycle, mutation of *Cry2* in MYC-transformed fibroblasts suppressed p53 and enhanced growth ( Chan *et al*., 2021) whereas downregulation of *BMAL1* and *CLOCK* in human glioblastoma stem cells induced cell cycle arrest and apoptosis ( Chen *et al*., 2020; Dong *et al*., 2019), demonstrating a cancer and tissue-specific effect of the clock on tumorigenesis. The circadian clock has also been shown to regulate immunity and metastasis as clock gene dysregulation is correlated with increased inflammation ( Gibbs *et al*., 2014) and T cell exhaustion ( Wu *et al*., 2019). Chronic jet lag promotes an immunosuppressive microenvironment, enhances stemness, and increases cancer cell metastasis ( Hadadi *et al*., 2020) and intravasation of circulating breast tumor cells was shown to have time-of-day frequency ( Diamantopoulou *et al*., 2022) suggesting potential clock-control of metastatic seeding. A direct link between circadian immune function and anti-tumor immunity was demonstrated by clock-dependent trafficking of DCs to the tumor draining lymph node regulating circadian function of tumor-antigen specific CD8s and melanoma volume after engraftment ( Wang *et al.*, 2022). Lastly, the circadian clock has been implicated in metabolic pathways involved in driving cellular proliferation, especially related to the crosstalk between the clock and MYC signaling ( Altman *et al*., 2015; Chun, Fortin, Fellows *et al*., 2022; Shostak *et al*., 2016; Stokes *et al*., 2021). Figure created using BioRender.

Although there is mounting evidence suggesting that the circadian clock is implicated in various types of cancer, the mechanism underlying the role of the clock in cancer is still being uncovered. Table 2 outlines significant findings that provide clues for how the circadian clock functions in various cancer types and model systems. Based on these studies, the circadian clock has been implicated in both the initiation and the progression of cancer through the regulation of oncogenic pathways, cell cycle control, DNA damage repair, stemness, immunity and metastasis ( Figure 2). However, the effect of clock disruption on tumorigenesis may be tissue and model-specific. For example, knock out of the core clock gene *BMAL1* in mouse models of solid tumors promotes tumor progression in CRC ( Chun, Fortin, Fellows *et al*., 2022; Stokes *et al*., 2021), lung ( Papagiannakopoulos *et al*., 2016), and other cancer types ( Lee *et al*., 2010) but reduces the development of cutaneous squamous tumors ( Janich *et al*., 2011). Furthermore, downregulation of *BMAL1* in human glioblastoma stem cells halts their growth ( Dong *et al*., 2019; Puram *et al*., 2016). As more studies are done in multiple cancer types and model systems, we may begin to better delineate the tissue-specific effects of clock disruption on cancer.

**Table 2.** Circadian clock function in cancer models.

Reference	Model system	Cancer type	Finding
( Shilts *et al*., 2018)	TCGA	Over 20 cancer types	Coordinated clock gene expression is lost in tumor vs non-tumor samples.
( Ye *et al*., 2018)	TCGA, cell lines	32 cancer types	Clock genes are associated with activation/inhibition of oncogenic pathways, mutations in core clock genes correlated with patient survival, and circadian rhythmicity is lost in cancer cell lines.
( Wu *et al*., 2019)	TCGA	11 cancer types	Core circadian clock genes are dysregulated in cancer and dysregulation correlated with poor patient prognosis and T cell exhaustion.
( Papagiannakopoulos *et al*., 2016)	Mouse	Lung	Genetic ( *Per2* ^m/m^ and *Bmal1* ^-/-^) and environmental (jet lag) clock disruption increased lung tumorigenesis in *K-ras ^LSL-G12D/+^;p53 ^flox/flox^* or *K-ras ^LSL-G12D/+^* (K) mice.
( Pariollaud *et al*., 2022)	Mouse and human	Lung	Environmental clock disruption through jet lag increased lung tumor burden in *Kras ^LSL-G12D/+^* and enhanced HSF1 signaling. Inhibition of HSF1 reduced the growth of human lung cancer cells.
( Lee *et al*., 2010)	Mouse	Lymphoma, osteosarcoma, liver, angiosarcoma, ovarian, uterine	*Per1 ^-/-^, Per2 ^-/-^, Cry1 ^-/-^, Cry2 ^-/-^* and *Bmal1* ^-/-^ mice presented with increased spontaneous and radiation-induced tumor development.
( Kettner *et al*., 2016)	Mouse	Liver	Chronic jet lag induced spontaneous HCC in WT mice.
( Fu *et al*., 2002)	Mouse	Liver	Gamma irradiation of *Per2 ^-/-^* mice caused increased tumor development and reduced apoptosis in thymocytes. Genes involved in cell cycle regulation and tumor suppression were deregulated in *Per2 ^-/-^* mice.
( Wood *et al*., 2008)	Mouse	Colorectal	*Per2 ^-/-^* mice developed colonic poylps and *Apc ^Min/+^/Per2 ^-/-^* developed significantly more intestinal polyps than *Apc ^Min/+^* mice.
( Stokes *et al*., 2021)	Mouse	Colorectal	*Apc ^Min/+^/Bmal1 ^-/-^* mice developed more intestinal polyps than *Apc ^Min/+^* mice.
( Chun, Fortin, Fellows *et al*., 2022)	Mouse and human tumors *ex vivo*	Colorectal	*Apc ^ex1-15^/Bmal1 ^-/-^* mice developed more intestinal polyps than *Apc ^ex1-15^* mice. Organoids from *Apc ^ex1-15^/Bmal1 ^-/-^* mice transformed into tumor spheroids due to *Apc* LOH. Circadian rhythms were lost in human colorectal tumors versus normal surrounding epithelial.
( Hadadi *et al*., 2020)	Mouse	Breast	Chronic jet lag increases cancer cell dissemination and lung metastasis, enhances stemness and promoted tumorigenesis by creating an immunosuppressive tumor microenvironment.
( Diamantopoulou *et al*., 2022)	Human, mouse	Breast	Intravasation of circulating breast tumor cells, which were prone to metastasize, occurred more frequently at night.
( Shafi *et al*., 2021)	*In vitro*, *in vivo* and human tumors *ex vivo*	Prostate	*CRY1* expression is correlated to poor patient survival. *CRY1* is stabilized by DNA damage in cancer and regulates homologous recombination.
( Chan *et al*., 2021)	TCGA and primary mouse fibroblasts	Bladder, colorectal, breast, stomach, melanoma, head and neck	*CRY2* is mutated in human bladder, colorectal, breast, stomach, melanoma, and head and neck cancers. *Cry2* mutation in MYC-transformed fibroblasts suppressed *p53* gene expression and enhances growth.
( Fekry *et al*., 2018)	Mouse	Liver	*Bmal1* expression in HNF4α-positive HCC prevented the growth of tumors *in vivo.*
( Sulli *et al*., 2018b)	Human *in vitro* and mouse *in vivo*	Multiple cancer types including colon, breast, melanoma and glioblastoma	Treatment of human cancer cells with SR9009, a REV-ERBα/β agonist, impaired viability and promoted apoptosis Treatment of glioblastoma in mice with SR9009 reduced growth, triggered apoptosis and improved survival
( Janich *et al*., 2011)	Mouse	Cutaneous squamous carcinoma	*Bmal1 ^-/-^/K5-SOS* mice developed fewer tumors than *Bmal1 ^+/+^/K5-SOS* mice.
( Dong *et al*., 2019)	Human *ex vivo*	Glioblastoma stem cells	Downregulating *BMAL1* and *CLOCK* induced cell cycle arrest and apoptosis. Small molecule agonists targeting Cryptochromes and REV-ERBs downregulated stem cell factors and reduced GSC growth.
( Chen *et al*., 2020)	Mouse	Glioblastoma	*CLOCK* enhanced stem cell self-renewal and promoted protumor immunity through *OLFM3* expression. *CLOCK* depletion in GSC272 and GSC20 tumors prior to implantation extended overall survival.
( Puram *et al*., 2016)	*In vitro* and *in vivo*	Acute myeloid leukemia	Inhibiting *Bmal1* in AML cells reduced self-renewal. *Bmal1 ^-/-^* AML cells exhibited a growth defect compared to *Bmal1 ^+/+^* AML cells. Irradiated WT mice transplanted with *Bmal1* ^-/-^ AML cells survived significantly longer than mice transplanted with *Bmal1* ^+/+^ AML cells.
( Altman *et al*., 2015)	Cell lines	Neuroblastoma	Overexpression of *Bmal1* suppressed colony formation.
( Moreno-Smith *et al.*, 2021)	Cell lines	Neuroblastoma	MYCN suppressed *BMAL1* expression to promote cell survival and this is attenuated by overexpression of *BMAL1* by SR1078 treatment.
( Shostak *et al*., 2016)	Cell lines	Osteosarcoma	Overexpression of *MYC* disrupted the clock and promoted proliferation.

## Highlighting the circadian clock and CRC

**Figure 3.**
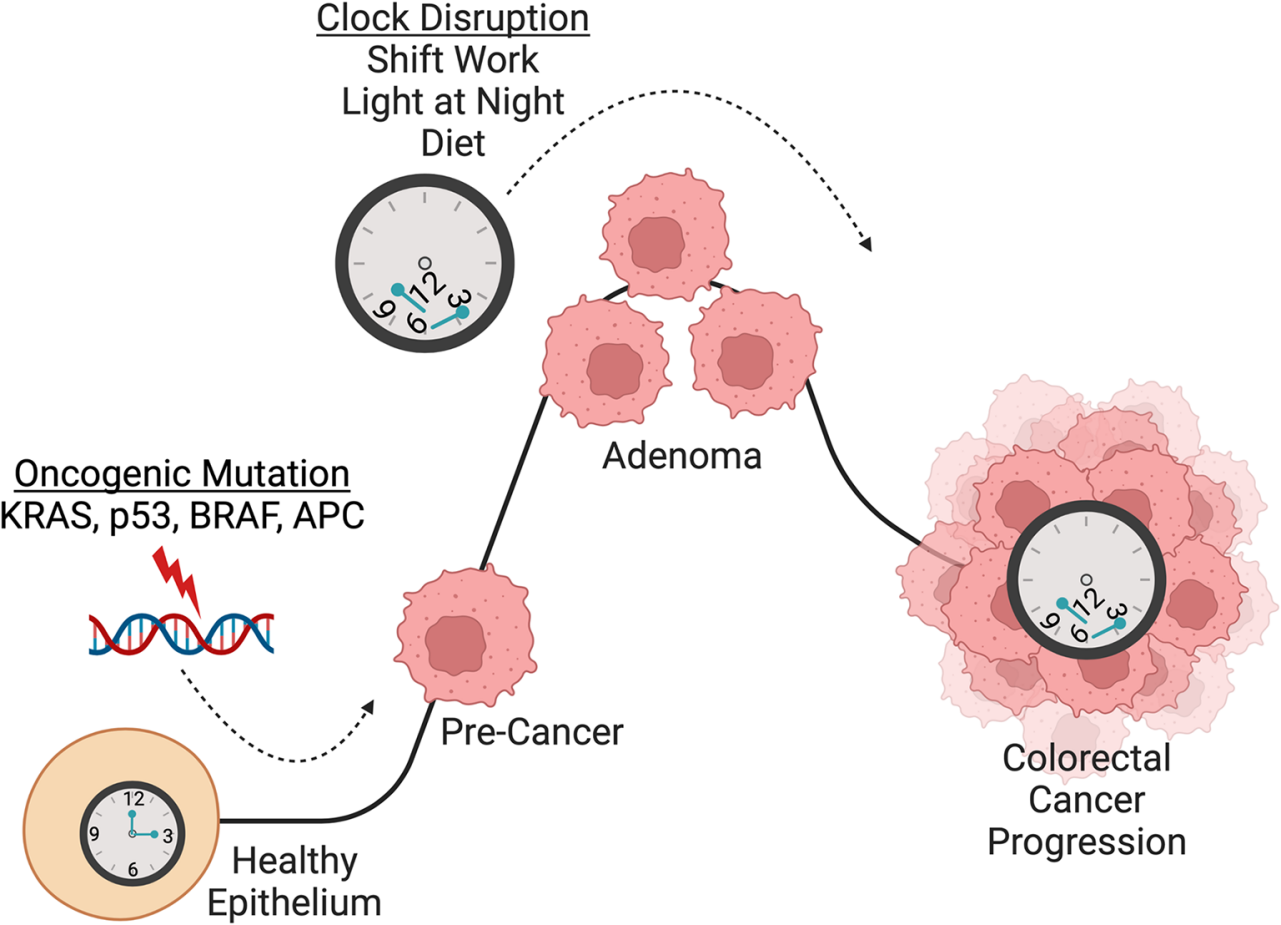
Circadian clock disruption as a driver of colorectal carcinogenesis. CRC has been shown to be initiated by sequential mutations in known cancer-causing genes including APC, KRAS, p53, and SMAD4 ( Drost *et al*., 2015; Li *et al*., 2014). Numerous studies have found that the circadian clock is involved in CRC initiation and progression. Importantly, circadian clock disruption promotes CRC pathogenesis in multiple mouse models ( Chun, Fortin, Fellows *et al*., 2022; Stokes *et al*., 2021; Wood *et al*., 2008). Additionally, the core clock gene CLOCK was found to be mutated in 53% of CRC that display microsatellite instability and it was shown that CLOCK binds near DNA damage related genes p21, BRCA1 and RAD50 to mediate DNA repair, apoptosis, and cell cycle arrest ( Alhopuro *et al*., 2010). Loss of *Bmal1* in *Apc ^ex1-15/+^* mice and intestinal organoids accelerated *Apc* LOH which drove transformation ( Chun, Fortin, Fellows *et al*., 2022). These studies implicate the circadian clock in maintenance of genome stability and demonstrate a role for circadian clock disruption in promoting colorectal carcinogenesis. An increasing number of studies have also explored the relationship between CRC and circadian clock disruption through shift work, light-at-night, and diet. Night shift work in humans has been shown to increase the risk of developing CRC ( Papantoniou *et al*., 2017; Schernhammer *et al*., 2003) and chronic jet lag, through exposure to light and night, increases CRC tumor burden in mice ( Chun, Fortin, Fellows *et al*., 2022; Stokes *et al*., 2021). High-fat diet also disrupts molecular circadian rhythms ( Eckel-Mahan *et al*., 2013; Hatori *et al*., 2012; Kohsaka *et al*., 2007). Given that HFD is known to enhance tumorigenicity of intestinal progenitors ( Beyaz *et al*., 2016; Mana *et al*., 2021) and exacerbate CRC ( Goncalves *et al*., 2019), the potential link with the intestinal clock warrants further investigation. Overall, compelling evidence implicates circadian clock disruption in CRC carcinogenesis, which suggests that night shift work, light-at-night, and diet could be potential drivers of CRC progression in humans, and especially in young-onset CRC. Figure created using BioRender.

As discussed in the previous section, there is a growing body of evidence suggesting a role for circadian clock disruption in various types of cancer. CRC is of particular interest as early-onset CRC is increasing at an alarming rate, faster than any other type of cancer ( Augustus & Ellis, 2018; Bailey *et al*., 2015; Muller *et al*., 2021; Singh *et al*., 2014). Circadian disruption due to *Bmal1* knockout was sufficient to drive CRC past the initiation stage ( Chun, Fortin, Fellows *et al*., 2022; Stokes *et al*., 2021). The importance of multiple signaling pathways in CRC progression including Wnt, TGF-β, Notch, EGFR/MAPK and PI3K has been reviewed previously ( Koveitypour *et al*., 2019). Here, we discuss how the circadian clock is disrupted in CRC and what molecular processes are governed by the clock that, when perturbed, result in CRC progression ( Figure 3).

### Wnt signaling is linked with the circadian clock

Wnt signaling is an important pathway for many processes including development, proliferation, and apoptosis ( Nusse & Clevers, 2017). This is especially relevant for the intestine, a highly regenerative organ where differentiated cells are replaced every four to five days by stem cells located in the crypt base ( Van Der Flier & Clevers, 2009). The Wnt pathway is upregulated when Wnt ligands bind the frizzled receptor and trigger inactivation of the destruction complex, composed of APC, GSK3-β and AXIN ( Neufeld *et al*., 2000; Orford *et al*., 1997; Rubinfeld *et al*., 1993; Su *et al*., 1993). As a result, β-catenin evades proteosome-dependent degradation, and shuttles into the nucleus to co-activate TCF-LEF mediated transcription of Wnt-dependent genes ( Hoverter *et al*., 2014; Molenaar *et al*., 1996).

The intestine is a highly rhythmic organ and rhythmicity is important in functions such as peristalsis, permeability and secretion of digestive enzymes ( Codoñer-Franch & Gombert, 2018). The molecular clock is involved in regulating intestinal circadian rhythms as core clock genes are expressed in many intestinal cell types including stem, progenitor, tuft, enteroendocrine and enterocytes ( Habowski *et al*., 2020). Circadian rhythms of intestinal stem cells (ISCs) are thought to be driven, at least in part, by Wnt signaling secreted from differentiated cells. ISC clocks were responsive to both Wnt and Hippo signaling in the stem cell niche ( Parasram *et al*., 2018). Furthermore, Paneth cells were found to rhythmically secrete Wnt and many Wnt pathway components have rhythmic expression ( Matsu-Ura *et al*., 2016; Soták *et al*., 2013). This is likely to be essential for proper ISC function as Wnt signaling was found to couple the clock and the cell cycle in the intestine ( Matsu-Ura *et al*., 2016) and the coupling of clock and cell cycle is conserved across multiple species ( Hong *et al*., 2014a; Yang *et al*., 2010). In the intestine, the molecular clock was found to gate cell cycle progression and was important for ISC regeneration after DSS induced damage ( Karpowicz *et al*., 2013; Matsu-Ura *et al*., 2016).

### The clock and aberrant signaling in CRC

The Wnt signaling pathway is highly mutated in human CRC with nearly all tumors containing inactivating mutations in *APC* or *GSK3*-β, or stabilizing mutations in *CTNNB1* (β-catenin) ( Kwong & Dove, 2009). Around 80% of sporadic human CRC contain a mutation in *APC* ( Fearnhead *et al.*, 2001). In many cases this is followed by mutation of the wild type allele, also known as loss of heterozygosity (LOH) ( Fearnhead *et al.*, 2001; Kwong & Dove, 2009). Due to its important role in the destruction complex, *APC* mutation results in aberrant activation of the Wnt signaling pathway ( Korinek, 1997; Morin, 1997; Moser *et al.*, 1990). *APC* mutations are sufficient to drive CRC initiation of adenoma growth ( Cheung *et al.*, 2010; Lamlum *et al.*, 2000; Rowan *et al.*, 2000; Zauber *et al.*, 2016; Zhang & Shay, 2017). However, secondary driver mutations in key genes such as *KRAS*, *TP53*, and *SMAD4* are required for progression to adenocarcinoma ( Drost *et al.*, 2015; Fearon & Vogelstein, 1990; Vogelstein *et al.*, 1988). The circadian clock has recently been identified as a secondary driver of CRC. *Bmal1* loss was found to promote *Apc* LOH by increasing genome instability and resulting in Wnt signaling hyperactivation in mice ( Chun, Fortin, Fellows *et al.*, 2022). Additionally, downregulation of *PER2* in human colon cancer cells HCT116 and HT29 increased β-catenin levels and cell proliferation ( Wood *et al.*, 2008). Increased β-catenin in CRC cell lines also enhanced *PER2* degradation by upregulating β-TrCP, an E3 ubiquitin ligase component ( Yang *et al.*, 2009). As *APC* is involved in regulation of cell-cell adhesion, microtubule stability, cell cycle and apoptosis ( Fearnhead *et al.*, 2001), clock disruption mediated *APC* LOH could perturb multiple pathways important in CRC progression.

Aside from Wnt signaling, other pathways have also been implicated in clock mediated acceleration of CRC. The Hippo pathway regulates multiple key processes including proliferation, differentiation, tissue growth, and regeneration. A cascade of serine/threonine kinases act to sequester Yes associated protein (YAP1) and transcriptional activator with PDZ binding motif (TAZ) in the cytoplasm and prevent them from activating the pro-survival TEA DNA binding (TEAD) family of transcription factors. Dysregulation results in an increase in YAP/TAZ which is associated with many human cancers, mediating increased proliferation and metastasis ( Calses *et al.*, 2019). In an *Apc* *^Min/+^* mutant mouse model, *YAP1* was found to be required for the progression of early initiating cells by suppressing differentiation and promoting regeneration ( Gregorieff *et al.*, 2015). The Hippo pathway may be involved in clock mediated acceleration of CRC. *Yap* and *Tead4* increased in *Apc* ^*Min/+*^; *Bmal1* ^*-/-*^ mice and were associated with increased self-renewal ( Stokes *et al.*, 2021). Furthermore, *Apc* ^ex1-15/+^; *Bmal1* ^-/-^ organoids had increased expression of YAP/TAZ pathway components compared to *Apc* ^ex1-15/+^ organoids ( Chun, Fortin, Fellows *et al.*, 2022). In summary, clock disruption accelerates the pathogenesis of CRC, and based on data from pre-clinical studies, the circadian clock likely impinges on several important signaling pathways that regulate intestinal biology.

### Clock disruption in CRC

Misregulation of molecular clock components have frequently been identified in human CRC. Multiple studies have reported decreased *BMAL1, CRY1-2 and PER1-3,* and increased *CLOCK*, *CSNK1E* and *TIM* in tumor tissue relative to matched healthy mucosa ( Hong *et al*., 2014b; Krugluger *et al*., 2007; Mazzoccoli *et al*., 2011, 2016; Oshima *et al*., 2011; Zeng *et al*., 2014). This was also linked to disease progression as reduced *BMAL1, PER1* or *PER3* expression was associated with poor overall survival ( Mazzoccoli *et al*., 2011; Zeng *et al*., 2014). Additionally, clock genes were found to be mutated in cancer and therefore might be involved in pathogenesis. A large fraction of CRC patients with microsatellite instability (MSI) had a mutation in *CLOCK* which could decrease *CLOCK* expression in MSI CRC cell lines ( Alhopuro *et al*., 2010; Mazzoccoli *et al*., 2011).

Pre-clinical genetic mouse models have also demonstrated that clock disruption accelerates CRC pathogenesis. In the azoxymethane and dextran sodium sulfate (AOM/DSS) model of colitis associated CRC, the circadian rhythmicity of *Per1*, *Per2*, *Reverb, Dbp* and *Bmal1* was reduced in tumors compared to healthy colon ( Soták *et al*., 2013). In an *Apc ^Min/+^* model of CRC, tumors exhibited reduced overall expression of key clock components *Rev-Erbα*, *Bmal1* and *Per2*, with complete loss of *Per2* rhythm ( Stokes *et al*., 2021; Yang *et al*., 2009). Furthermore, clock disruption was found to increase tumor burden when *Per2* or *Bmal1* were deleted in *Apc ^Min/+^* mice ( Stokes *et al*., 2021; Wood *et al*., 2008). In a novel mouse model, where exons 1 to 15 in *Apc* were deleted in one allele ( *Apc ^ex1-15/+^*), *Bmal1* knockout increased tumor incidence, enlarged polyps and decreased survival ( Chun, Fortin, Fellows *et al*., 2022). Additionally, environmental circadian disruption through use of a light shift paradigm or constant light increased both polyp formation and size of tumors in *Apc ^ex1-15/+^* and *Apc ^min/+^* mice ( Chun, Fortin, Fellows *et al*., 2022; Stokes *et al*., 2021). Together these results suggest that circadian disruption can play a key role in driving pathogenesis of CRC.

## Circadian clock and prevention/treatment of cancer

In modern society, the necessity of night shift work and the presence of artificial light at night warrants a better understanding of the impact of circadian disruption on health and disease. Above, the literature defining circadian clock function in critical cellular processes and the role of circadian clock disruption in various cancer types was summarized. In this section, emerging ideas for how the circadian clock can be leveraged to both prevent and treat cancer are highlighted. For additional information on this topic, a more extensive review of chronotherapeutic approaches has recently been published ( Sulli *et al*., 2018a).

### Disease prevention approaches through the lens of the clock

Promoting robust circadian rhythms through consistent sleep and feeding behavior is an important regulator of physiological health. However, night shift workers are faced with irregular activity-rest and feeding-fasting rhythms as well as artificial light at night exposure, all of which are known to disrupt the circadian clock. Key literature was reviewed above that aimed to define the correlation between night shift work and cancer prevalence. Although this body of literature requires more comprehensive studies to draw definitive conclusions, the importance of proper alignment of circadian rhythms has emerged as a key theme. Numerous studies cite a significant increase in cancer risk after long-term night shift work, typically 15–20 years ( Åkerstedt *et al*., 2015; Hansen & Lassen, 2012; Wegrzyn *et al*., 2017). Risk was also seen to increase with duration of exposure ( Davis *et al*., 2001; Hansen & Lassen, 2012; Hansen & Stevens, 2012; Lie *et al*., 2011). For example, in nurses who worked night shift for over five years, the risk of developing breast cancer increased from an odds ratio of 1.4 to 1.8 with increasing consecutive night shifts ( Lie *et al*., 2011). In order to reduce the risk of cancer in night shift workers, the consecutive duration of night shifts may need to be limited as well as the cumulative exposure to night shift work.

Circadian misalignment, defined as a disruption or misalignment between an individual’s internal circadian clock and the external cues such as light-dark and feeding-fasting cycles, is an unavoidable consequence of night shift work. However, recent research has recommended lifestyle interventions as a means of combating these effects. Night shift workers often disrupt their feeding-fasting patterns which has been shown to disrupt glucose metabolism ( Spiegel *et al*., 1999). Night shift work has also been significantly associated with metabolic syndrome ( Wang *et al*., 2014), which increases the risk for developing various types of cancer ( Esposito *et al*., 2012). Therefore, increasing metabolic health through lifestyle intervention may combat the increased risk of cancer. One such intervention is time-restricted eating (TRE), which involves limiting the eating window to 6–12 hours per day ( Manoogian *et al*., 2022). In a study with prediabetic men, limiting the feeding window to six hours for five weeks improved insulin sensitivity, blood pressure, oxidative stress, and appetite ( Sutton *et al*., 2018). A recent study found that a 10-hour feeding window in 24-hour shift workers is a feasible intervention to reduce weight, improve cardiometabolic health, sleep quality, and mood ( Manoogian *et al*., 2022).

It has been well established that night shift is associated with circadian misalignment, however, the general population is increasingly at risk of circadian misalignment through inconsistent eating patterns and artificial light-at-night exposure. Therefore, it may be beneficial to update cancer screening measures ( Patel *et al*., 2022; Wolf *et al*., 2018). As the percentage of early-onset cancer increases, the screening age should decrease accordingly for early detection and prevention measures. Promoting robust circadian rhythms through consistent sleep, feeding, regulation of night shift work, and lifestyle interventions such as TRE may help improve parameters that impinge on human health and could offset the impacts of circadian misalignment on specific cancer types.

### Cancer chronomedicine

Cancer chronotherapy refers to the timing of an anticancer drug to increase efficacy and decrease toxicity. This approach is based on the rationale that the drug will be better tolerated at certain times of day based on the mechanism of action of the drug. A recent comprehensive review of chronomodulated chemotherapy has recently been reported ( Printezi *et al*., 2022). In this systematic review, 11 of 18 studies found that chronomodulated chemotherapy significantly decreased toxicity while maintaining efficacy. More specifically, chronomodulated chemotherapy reduced side effects including nausea, vomiting, mucositis and leukopenia for nasopharyngeal carcinoma ( Gou *et al*., 2018; Zhang *et al*., 2018), breast ( Coudert *et al*., 2008), colorectal ( Lévi *et al*., 1994, 1997) and endometrial cancer ( Gallion *et al*., 2003). Reducing side effects is a critical aspect of patient care as it improves quality of life and often allows for higher or more frequent doses. Although chronomodulated chemotherapy does appear to reduce side effects, it remains elusive whether chronotherapy improves drug efficacy or prognosis. For example, only three studies report higher response rate and longer survival in chronotherapy treated groups ( Gou *et al*., 2018; Lévi *et al*., 1994, 1997). However, two of the three of these studies report higher dose intensity in the chronotherapy treated group because of the reduced side effects. Therefore, it is unclear whether improved response is due to increased dose or a direct result of chronotherapy. A meta-analysis on factors impacting drug timing effects found that study size and whether or not the study was publicly registered as a clinical trial affected the reported efficacy of chronomedicine ( Ruben *et al*., 2021), suggesting that further studies are needed. In summary, optimal timing of anticancer drugs appears to reduce toxicity but more mechanistic studies are needed to determine the clinical relevance of anticancer chronotherapeutic approaches on drug efficacy.

## Concluding remarks and future directions

The circadian clock is an evolutionarily conserved internal timekeeping system that maintains homeostasis within the body. In this review, we discussed the connection between the circadian clock and critical biological processes including cell cycle control, DNA damage response, DNA repair, and immunity. Disruption of these processes are known hallmarks of cancer ( Hanahan, 2022; Hanahan & Weinberg, 2000, 2011), and we highlight the links between circadian clock disruption and cancer through clinical, epidemiological, and pre-clinical molecular studies. Though progress has been made to deconvolute the role of the circadian clock in cancer, this review highlights the divergent evidence linking circadian clock disruption with tumorigenesis. For clinical and epidemiological studies, these differing conclusions may be due to self-reporting, confounding factors, and non-standardized definitions of night shift work. For molecular and mechanistic studies, clock-controlled rhythmic expression is known to be tissue-specific, suggesting that the impact of circadian clock disruption would also be tissue specific. This makes drawing a simplified conclusion regarding the role of clock disruption on tumorigenesis difficult. Future studies are needed to systemically explore these tissue-specific differences and determine the role of clock disruption in each organ independently. These more comprehensive studies will yield a foundational understanding by which the circadian clock can be leveraged for cancer prevention and chronomedicine-based approaches.

Finally, with the alarming rise in the rate of early-onset cancers and the necessity of night shift work in modern society, it is imperative to address the concern of circadian clock disruption in a growing population of individuals afflicted by circadian misalignment. In this review, we highlighted how individuals can promote healthy circadian rhythms by limiting the exposure to night shift work, lifestyle interventions such as TRE, and updated cancer screening. This list is not comprehensive and additional molecular studies are needed to guide our understanding of intervention approaches that can offset circadian clock disruption. Overall, the circadian clock presents a unique and underexplored connection between health and disease which has the potential for therapeutic value in cancer treatment.

## Data Availability

No data are associated with this article.
