# Supplementary material for: Circadian clocks in health and disease: Dissecting the roles of the biological pacemaker in cancer
Source: F1000Res. 2023 May 16;12:116. Originally published 2023 Feb 1. [Version 2] doi: 10.12688/f1000research.128716.2 (PMC11399774; doi:10.12688/f1000research.128716.2)
Supplement: Peer review history

# Reviewer report: Reviewer response for version 2

**DOI:** 10.5256/f1000research.147484.r173741

**Published:** 2024-09-13

**Authors:** Phillip Karpowicz

**Affiliations:** 1 Department of Biomedical Sciences, University of Windsor, Windsor, ON, Canada

The authors have responded to all my questions / concerns.

Is the review written in accessible language?

Yes

Are all factual statements correct and adequately supported by citations?

Yes

Are the conclusions drawn appropriate in the context of the current research literature?

Yes

Is the topic of the review discussed comprehensively in the context of the current literature?

Yes

Reviewer Expertise:

Circadian rhythms, stem cells

I confirm that I have read this submission and believe that I have an appropriate level of expertise to confirm that it is of an acceptable scientific standard.

# Reviewer report: Reviewer response for version 1

**DOI:** 10.5256/f1000research.141333.r162021

**Published:** 2023-03-02

**Authors:** Kristin Eckel-Mahan

**Affiliations:** 1 Institute of Molecular Medicine, McGovern Medical School, University of Texas Health Science Center, Houston, TX, USA

In 'Circadian clocks in health and disease: Dissecting the roles of the biological pacemaker in cancer', Fortin *et al.* provide a brief but well-balanced review of key molecular mechanisms discovered to date that are involved in the links between circadian clock disruption and cancer. The figures are nicely done and the tables are very helpful in summarizing the information. Overall, this is a nicely written article and I have only a few suggestions for improvement.

1. Considering the emphasis on early-onset cancers in the abstract, the authors may wish to draw a tighter connection between clock disruption and early-onset cancers in the manuscript. The authors give a nice molecular summary of CRC, the prevalence of which seems to be particularly pronounced in younger populations. The authors may wish to discuss more why CRC might show a tighter link to clock function than some of the other tumor types. In the context of CRC, the authors may want to add that intestinal tumors were also found in the Lee *et al. *(2010) study that they cite.
2. I realize that the review does not focus on neuroblastoma; however, considering the discussion regarding BMAL1 and MYC, the authors may wish to cite Moreno-Smith *et al. *(2021) ^1^, where MYC downregulates BMAL1 in MYCN-positive neuroblastomas and suppresses clock function.
3. In the paragraph discussing base excision repair, it would nice if the authors would provide additional details about the studies showing clock regulation of the enzyme OGG1 (is it tissue-specific, observed *in vivo* vs. *in vitro*, etc.?), especially considering the interesting links to shift work.
4. When covering the Keller *et al.* (2009) paper discussing the role of the clock in splenic macrophages, the authors may wish to add that, like other cell types/tissues, much of the macrophage transcriptome is highly dynamic over the 24-hr. period.

Is the review written in accessible language?

Yes

Are all factual statements correct and adequately supported by citations?

Yes

Are the conclusions drawn appropriate in the context of the current research literature?

Yes

Is the topic of the review discussed comprehensively in the context of the current literature?

Yes

Reviewer Expertise:

Circadian rhythms

I confirm that I have read this submission and believe that I have an appropriate level of expertise to confirm that it is of an acceptable scientific standard.

## References

[1] : Restoration of the molecular clock is tumor suppressive in neuroblastoma. *Nat Commun* .2021;12(1) : 10.1038/s41467-021-24196-4 4006 10.1038/s41467-021-24196-4 34183658 PMC8238982

# Response

**Published:** 2023-04-28

**Authors:** Selma Masri

**Affiliations:** University of California, Irvine, USA

**Response to Reviewer Comments**

 Fortin et al., Circadian clocks in health and disease: Dissecting the role of the biological pacemaker in cancer

 F1000Research

**Reviewer #2**

*In 'Circadian clocks in health and disease: Dissecting the roles of the biological pacemaker in cancer', Fortin et al. provide a brief but well-balanced review of key molecular mechanisms discovered to date that are involved in the links between circadian clock disruption and cancer. The figures are nicely done and the tables are very helpful in summarizing the information. Overall, this is a nicely written article and I have only a few suggestions for improvement.*

 We appreciate the insightful feedback from Reviewer #2 and we are pleased that this reviewer finds our study “helpful” and “nicely written”. Below we have made every effort to address these valuable comments. 

*Considering the emphasis on early-onset cancers in the abstract, the authors may wish to draw a tighter connection between clock disruption and early-onset cancers in the manuscript. The authors give a nice molecular summary of CRC, the prevalence of which seems to be particularly pronounced in younger populations. The authors may wish to discuss more why CRC might show a tighter link to clock function than some of the other tumor types. In the context of CRC, the authors may want to add that intestinal tumors were also found in the Lee et al. (2010) study that they cite.*

 The reviewer is correct that CRC incidence is on the rise in younger populations. CRC may show a tighter link to clock function due to the following reasons which we have expanded upon in the text (pg 9).

- Intestinal epithelial cells are highly regenerative and this regeneration is regulated by the circadian clock. Thus, the intestine is especially sensitive to disruption of circadian rhythms which can affect the coordination of cellular turnover (Stokes *et al., *CMGH; Yoshida *et al.,* Chronobiol Int; Codoñer-Franch *et al.,* WJG).  
- The gut microbiota exhibits circadian rhythmicity and is negatively impacted by circadian disruption (Liang *et al.,* PNAS; Heddes *et al.,* Nat Commun).
- Alterations of the circadian clock, through timing of food intake and high fat diet, can disrupt the circadian clock in the intestine (Eckel-Mahan *et al.,* Cell; Damiola *et al*., Genes Dev; Chaix *et al.,* Cell Metab).

*I realize that the review does not focus on neuroblastoma; however, considering the discussion regarding BMAL1 and MYC, the authors may wish to cite Moreno-Smith et al. (2021) 1, where MYC downregulates BMAL1 in MYCN-positive neuroblastomas and suppresses clock function.*

 We thank the reviewer for bringing up this study and have included the study in Table 2 as well as the text (pg 5).

*In the paragraph discussing base excision repair, it would nice if the authors would provide additional details about the studies showing clock regulation of the enzyme OGG1 (is it tissue-specific, observed in vivo vs. in vitro, etc.?), especially considering the interesting links to shift work.*

 This is an important point. The regulation of OGG1 has not been extensively studied. However, while one study found OGG1 to oscillate in both *ex vivo* and *in vitro* human lymphocytes (Manzella *et al.,* Sci Rep), another found no such oscillation in human keratinocytes (Hettwer *et al.,* Int J Cosmet Sci). This suggests a potential cell-type specific role of the circadian clock is regulating OGG1 expression. We have addressed these points in the text (pg 6).

*When covering the Keller et al. (2009) paper discussing the role of the clock in splenic macrophages, the authors may wish to add that, like other cell types/tissues, much of the macrophage transcriptome is highly dynamic over the 24-hr. period.*

 We agree with the reviewer and have emphasized this point in the text (pg 7).

# Reviewer report: Reviewer response for version 1

**DOI:** 10.5256/f1000research.141333.r162020

**Published:** 2023-02-16

**Authors:** Phillip Karpowicz

**Affiliations:** 1 Department of Biomedical Sciences, University of Windsor, Windsor, ON, Canada

The authors review and discuss a growing body of literature that has explored connections between circadian rhythm disruption and cancer. This is now a long-standing and growing field, thus the review is helpful in updating the reader on the many new research papers that have been recently published. The authors highlight new advances in the field, particularly in the effect of rhythm disruption in colorectal cancer. The review is well-written and well-cited, the sections covering epidemiological studies as well as cellular and molecular mechanisms are covered thoroughly. The tables help summarize many studies, and particularly Table 2 is a good resource.

 I have only the following minor comments/suggestions:

1. Intro - The authors describe many studies which have found links between circadian clock genes and cell cycle regulators. In the intro (pg. 3), they might include a sentence mentioning that many of these studies were done in different tissues, and that the connections are tissue- or cell-specific. In addition, many studies have shown mechanisms occurring at the protein level rather than from the TTFL. For instance, the connections between Cry and cMyc, or Per and p53. The authors describe this area well and mention these issues but pg. 3 and Fig. 1 really only show the TTFL. The intro does not seem to reflect the complexity and themes covered in the review. Clarifying that clock genes can have cell-specific roles, and protein-based mechanisms aside from their role as transcriptional regulators would strengthen the intro.
2. In several parts of the manuscript, the authors mention the term “ *circadian misalignment*” but don’t really define it. A couple of sentences defining what this means would help clarify (do the authors mean molecular clock misalignment, individual cell re-entrainment, or inter-tissue misalignment, or all three?).
3. Figure 3 - With the finding that Bmal1 loss of function increases LOH, wouldn’t clock disruption be operating at the level of healthy epithelium/pre-cancer transition (where the Kras, APc, etc. mutation takes place)? The figure could indicate this contribution earlier than showing it at the stage past the pre-cancer step.
4. Page 14 – the authors might put *'Clock disruption in CRC'* section after *'The clock and aberrant signaling in CRC'* section – to first explain the role of Apc in colorectal cancer before discussing the pre-clinical mouse models (Apc ex1-15 and Apc min). This would also fit better right after discussion of the Wnt pathway in the intestine.

Is the review written in accessible language?

Yes

Are all factual statements correct and adequately supported by citations?

Yes

Are the conclusions drawn appropriate in the context of the current research literature?

Yes

Is the topic of the review discussed comprehensively in the context of the current literature?

Yes

Reviewer Expertise:

Circadian rhythms, stem cells

I confirm that I have read this submission and believe that I have an appropriate level of expertise to confirm that it is of an acceptable scientific standard, however I have significant reservations, as outlined above.

# Response

**Published:** 2023-04-28

**Authors:** Selma Masri

**Affiliations:** University of California, Irvine, USA

**Response to Reviewer Comments**

 Fortin et al., Circadian clocks in health and disease: Dissecting the role of the biological pacemaker in cancer

 F1000Research

**Reviewer #1**

*The authors review and discuss a growing body of literature that has explored connections between circadian rhythm disruption and cancer. This is now a long-standing and growing field, thus the review is helpful in updating the reader on the many new research papers that have been recently published. The authors highlight new advances in the field, particularly in the effect of rhythm disruption in colorectal cancer. The review is well-written and well-cited, the sections covering epidemiological studies as well as cellular and molecular mechanisms are covered thoroughly. The tables help summarize many studies, and particularly Table 2 is a good resource.*

 We thank Reviewer #1 for their comments regarding the quality of our work and have addressed each of these comments in the text and highlighted the changes below.

- *Intro - The authors describe many studies which have found links between circadian clock genes and cell cycle regulators. In the intro (pg. 3), they might include a sentence mentioning that many of these studies were done in different tissues, and that the connections are tissue- or cell-specific. In addition, many studies have shown mechanisms occurring at the protein level rather than from the TTFL. For instance, the connections between Cry and cMyc, or Per and p53. The authors describe this area well and mention these issues but pg. 3 and Fig. 1 really only show the TTFL. The intro does not seem to reflect the complexity and themes covered in the review. Clarifying that clock genes can have cell-specific roles, and protein-based mechanisms aside from their role as transcriptional regulators would strengthen the intro.*

This is an important point, and we are happy that the reviewer raised this issue. In the introduction (pg 3) and in Figure 1, we have highlighted the role of the circadian clock being cell- and tissue-specific as well as circadian regulation occurring at both the transcriptional and protein level.

- *In several parts of the manuscript, the authors mention the term “circadian misalignment” but don’t really define it. A couple of sentences defining what this means would help clarify (do the authors mean molecular clock misalignment, individual cell re-entrainment, or inter-tissue misalignment, or all three?).*

We refer to “circadian misalignment” as misalignment between an individual’s internal circadian clock and the external cues such as light-dark and feeding-fasting cycles, and have now been defined in the text (pg 15).

- *Figure 3 - With the finding that Bmal1 loss of function increases LOH, wouldn’t clock disruption be operating at the level of healthy epithelium/pre-cancer transition (where the Kras, APc, etc. mutation takes place)? The figure could indicate this contribution earlier than showing it at the stage past the pre-cancer step.*

This is a critical point. Our work and others have shown that circadian clock disruption alone does not induce tumor initiation (Stokes *et al.,* Cell Mol Gastroenterol Hepatol; Chun, Fortin, Fellows *et al.,* Sci Adv). However, other studies have shown that clock disruption alone does initiate tumorigenesis, through indirect mechanisms (Lee *et al.*, PLoS One; Kettner *et al.*, Cancer Cell). Importantly, Kettner *et al. *demonstrates that clock disruption initiates hepatocellular carcinoma through non-alcoholic fatty liver disease, which is known to predispose to tumorigenesis. Lee *et al.* aged clock disrupted mice before identifying cancer initiation, making it difficult to disentangle the effect of clock disruption on an aging phenotype versus the effect on cancer initiation. Therefore, we feel that clock disruption alone is difficult to link to cancer initiation in either of these studies. In the majority of studies, it is only when cancer has initiated that clock disruption progresses tumorigenesis. For example, mice with genetic or environmental clock disruption are exposed to gamma irradiation (Fu *et al.*, Cell; Lee *et al.*, PLoS One), inoculated with tumor cells (Filipski et al., Cancer Res) or crossed with a tumor-initiating mutation (Lee *et al.,* PLoS One; Papagiannakopoulos *et al., *Cell Met; Pariollaud *et al.,* Sci Adv; Wood *et al*., MCR) to observe the effect of clock disruption on tumor progression. This suggests that clock disruption may not be sufficient to initiate cancer but instead is sufficient to progress cancer. Therefore, with the lack of sufficient evidence suggesting that clock disruption functions at the level of cancer initiation, we have placed it after the pre-cancer step in the figure. We also discuss in the review the function of the circadian clock in maintaining stemness and regeneration in normal intestinal epithelium, and these elegant studies are discussed in detail.

- *Page 14 – the authors might put 'Clock disruption in CRC' section after 'The clock and aberrant signaling in CRC' section – to first explain the role of Apc in colorectal cancer before discussing the pre-clinical mouse models (Apc ex1-15 and Apc min). This would also fit better right after discussion of the Wnt pathway in the intestine.*

We thank the reviewer for pointing this out. We agree with the reviewer on the order of these paragraphs and have made this change in the text.
